# Host–parasite fluctuating selection in the absence of specificity

**DOI:** 10.1098/rspb.2017.1615

**Published:** 2017-11-01

**Authors:** Alex Best, Ben Ashby, Andy White, Roger Bowers, Angus Buckling, Britt Koskella, Mike Boots

**Affiliations:** 1 School of Mathematics and Statistics, University of Sheffield, Sheffield S3 7RH, UK; 2 Department of Mathematical Sciences, University of Bath, Claverton Down, Bath BA2 7AY, UK; 3 Department of Integrative Biology, University of California Berkeley, Berkeley, CA, USA; 4 Department of Mathematics and the Maxwell Institute for Mathematical Sciences, Heriot-Watt University, Edinburgh EH14 4AS, UK; 5 Department of Mathematical Sciences, Division of Applied Mathematics, The University of Liverpool, Mathematical Sciences Building, Liverpool L69 7ZL, UK; 6 Biosciences, College of Life and Environmental Sciences, University of Exeter, Cornwall Campus, Treliever Road, Penryn, Cornwall TR10 9EZ, UK

**Keywords:** coevolution, infectious disease, fluctuating selection, specificity

## Abstract

Fluctuating selection driven by coevolution between hosts and parasites is important for the generation of host and parasite diversity across space and time. Theory has focused primarily on infection genetics, with highly specific ‘matching-allele’ frameworks more likely to generate fluctuating selection dynamics (FSD) than ‘gene-for-gene’ (generalist–specialist) frameworks. However, the environment, ecological feedbacks and life-history characteristics may all play a role in determining when FSD occurs. Here, we develop eco-evolutionary models with explicit ecological dynamics to explore the ecological, epidemiological and host life-history drivers of FSD. Our key result is to demonstrate for the first time, to our knowledge, that specificity between hosts and parasites is not required to generate FSD. Furthermore, highly specific host–parasite interactions produce unstable, less robust stochastic fluctuations in contrast to interactions that lack specificity altogether or those that vary from generalist to specialist, which produce predictable limit cycles. Given the ubiquity of ecological feedbacks and the variation in the nature of specificity in host–parasite interactions, our work emphasizes the underestimated potential for host–parasite coevolution to generate fluctuating selection.

## 1. Introduction

Understanding the coevolution of hosts and parasites is important given the central role that infectious disease plays in human health, agriculture and natural systems. Theory predicts that the coevolution of hosts and their parasites may lead to a number of distinct outcomes, including a coevolutionary stable strategy (co-ESS) for both host and parasite [1,2]; static within-population dimorphism or polymorphism [2–5]; escalation (known as arms race dynamics, ARD) [6] and fluctuating selection dynamics (FSD) [7–11]. ARD cannot continue indefinitely because of associated fitness costs or physiological constraints (e.g. [12]), which means that, in the long term, coevolution will eventually lead to either a stable evolutionary equilibrium (including polymorphisms) or fluctuating selection. Fluctuating selection is therefore of particular importance because it is the only dynamic coevolutionary outcome that can be maintained indefinitely in a constant environment. The presence of a constantly changing antagonist is thought to play a key role in the maintenance of diversity [13] and also has implications for selection for sex and recombination [14–16], and local adaptation [17–19]. Understanding the processes and mechanisms that promote FSD therefore has significant implications for our understanding of a wide range of biological phenomena.

Theoretical work has primarily focused on how different forms of genetic specificity between hosts and parasites lead to fluctuating selection [7–11,19–21]. Highly specific ‘matching-allele’ interactions, where parasites must ‘match’ the host at each loci to infect, commonly generate coevolutionary ‘cycles’ (i.e. FSD) as selection favours parasite genotypes capable of infecting common host genotypes, thereby generating negative frequency-dependent selection [20–22]. Effectively, hosts constantly evolve to ‘escape’ parasites that can infect them while parasites play ‘catch-up’. By contrast, ‘gene-for-gene’ interactions (where there is variation in specificity such that hosts and parasites vary from specialists to generalists) often produce ARD, where directional selection favours increasing resistance and infectivity ranges, although there can be a transition to FSD if there are costs to infection and defence [7–11]. While some empirical evidence appears consistent with the notion that different genetic interactions are associated with ARD or FSD [23–25], recent experimental work has shown that changing environmental conditions can cause host–parasite interactions to switch between ARD and FSD [26–28], suggesting either that the environment determines specificity or that the same genetic specificity has different consequences depending on the environment.

One way to investigate the importance of genetic specificity alongside ecological feedbacks in determining FSD is to directly compare coevolutionary dynamics with no specificity with those generated under various different forms of specificity. This can be achieved using eco-evolutionary models, which allow for varying population sizes owing to changes in host defence and parasite infectivity. These models are increasingly used to examine the role of environmental and ecological feedbacks on the coevolution of hosts and parasites [1,2,4,5,29] and have largely considered the processes that determine co-ESS levels of host defence and parasite infectivity, and the potential for diversification through evolutionary branching. For example, it has been shown that the likelihood of static, within-population diversification depends on the nature of host–parasite genetic specificity, associated fitness costs and explicit ecological dynamics [5]. The form of the infection interaction was crucial to the level of diversity that could arise, with non-specific ‘universal’ functions (parasite A always has higher transmission than parasite B against any host) leading to dimorphism at most, but ‘range’ functions with variation in specificity (whereby the relative success of parasite strains depends on the target host) potentially leading to higher levels of polymorphism [5]. This work emphasized the important role that ecological feedbacks play in host–parasite coevolution. Little of this work, however, has considered the potential for fluctuating selection [4,27], and none has provided a full exploration of the ecological, epidemiological and host life-history drivers of FSD.

Here, we examine how host and parasite life-history characteristics and the specificity of their interaction, in combination with ecological feedbacks, determine the likelihood of fluctuating selection. By ‘specificity’, we mean the degree to which parasite strains specialize on a subset of host types. An interaction is defined to be ‘specific’ if each parasite strain has higher transmission against some hosts and lower transmission against others compared with another parasite strain. Conversely, an interaction is ‘non-specific’ if each parasite strain always has either higher or lower transmission against all hosts compared with another parasite strain. We consider interactions between hosts and parasites starting from ‘universal’ (all non-specific) to ‘range’ (variation from highly specific to generalist, and therefore phenotypically equivalent to gene-for-gene models but with continuous phenotypic variation), and ‘matching’ (highly specific, where all parasite strains are specialists on respective host strains, and therefore phenotypically equivalent to matching-allele models but again with continuous phenotypic variation). Furthermore, we explicitly consider the ecological and epidemiological settings that promote cycles. As such, we determine what factors and which types of host–parasite interactions promote fluctuating selection.

## 2. Model and methods

We base our mathematical analysis within the eco-evolutionary invasion framework known as adaptive dynamics [30–33] and combine this with explicit evolutionary simulations that relax some of the restrictive assumptions of the mathematical approach (see the electronic supplementary material, §A1 for a fuller description of the analytical methods and the electronic supplementary material, §B for a description of the numerical simulations). We assume that resident strains of host and parasite have reached a population dynamic equilibrium of a susceptible–infected–susceptible model [5,34]:2.1
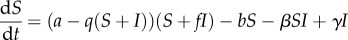
and2.2
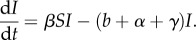


Susceptible hosts reproduce at a rate *a*, with the rate for infected hosts reduced by 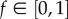, with reproduction limited by competition by a density-dependent factor *q*. All hosts die at a natural mortality rate *b*, but infected hosts suffer additional mortality at a rate *α*, which we define as ‘virulence’ (in contrast to the plant–pathogen literature where virulence is often defined as infectivity). Transmission is assumed to be a mass action density-dependent interaction with coefficient *β*. We assume that both the host and parasite have some ‘control’ over the transmission rate, so that transmission is dependent on the host trait, *h*, and parasite trait *p*, with 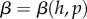. We will generally define *h* as susceptibility (i.e. inversely, resistance) and *p* as infectivity. Finally, hosts can recover from infection at a rate *γ*. For our algebraic analysis, we will make the simplifying assumptions that *γ* = 0 and *f* = 0, but we shall relax these assumptions in our numerical investigations.

We assume that a resident host (*h*) and parasite (*p*) are at their endemic steady state and that a rare mutant strain of either the host (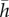; overbars denoting mutant traits) or parasite (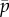) attempts to invade (with trait values limited to 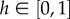 and 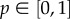 by some physiological constraints). The mutant has small phenotypic differences to the current resident strain and therefore a different transmission coefficient. We assume trade-offs in which a decrease in transmission (either an absolute reduction or an increase in resistance range; see below) caused by a host mutation confers a cost to the host birth rate, *a*(*h*), while an increase in the base transmission rate caused by a parasite mutation confers either an increase in virulence, *α*(*p*), or a reduced infection range [4,5]. The success of the mutant depends on its invasion fitness when the resident is at its ecological equilibrium. In the simplified case, where *γ* = 0, *f* = 0 (see the electronic supplementary material, §A1 for general case), the respective host and parasite fitnesses are2.3
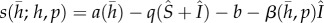
and2.4
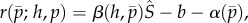
where hats denote equilibrium densities of the resident. If a mutant has positive invasion fitness, it will invade to replace or coexist with the current resident (subject to demographic stochasticity [30]), whereas if it has negative fitness, it will die out. Through a series of mutations and substitutions, the two species will co-evolve in the directions of their local selection gradients, with the canonical equations [30,31] given by2.5
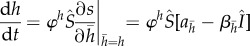
and2.6
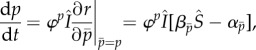
where subscripts denote derivatives (i.e. 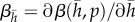) and *φ^i^* controls the respective speeds of mutation (which are products of the mutation rate and variance and a factor of 1/2). To simplify what follows, we shall set 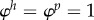. Note that all the derivatives are evaluated at the resident trait values, 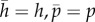.

A coevolutionary ‘singular point’ is a point at which the two selection gradients are simultaneously zero (i.e. there is no longer directional selection on either species). There are four behaviours at a singular point that are of particular interest. First, the singular point can be a long-term attractor of evolution (*continuously stable strategy* (CSS); a dynamic counterpart to the classic evolutionarily stable strategy (ESS)). Second, the singular point can be an *evolutionary branching point* for that species. Here, one of the species will undergo disruptive selection and branch into two coexisting strains. Third, if varying parameter values causes the system to pass a critical point (a Hopf bifurcation, [35]), then *coevolutionary cycles* will result (although further work is required to find whether the resulting cycles are stable, resulting in FSD, or unstable, resulting in bistability). Finally, a repelling singular point could cause directional selection in the host and/or parasite to *maximize or minimize* their investment to bounds of evolution (recall 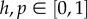), while the other species may reach a purely evolutionary CSS (i.e. a host CSS may exist where *p* *=* *1*), may branch, or may also *maximize*/*minimize*.

**Figure 1.**
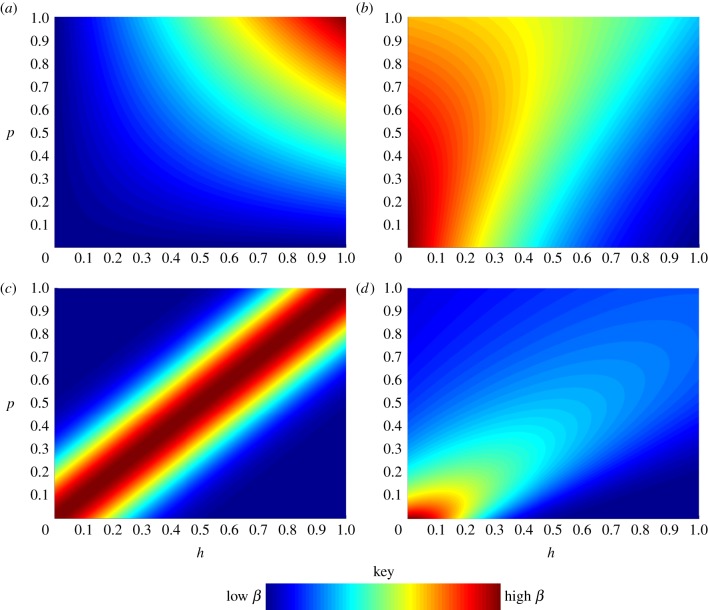
Heat maps showing the level of transmission, *β*, of parasite strains, *p*, against host strains, *h*, for our key infection functions: (*a*) universal, (*b*) range, (*c*) matching without costs, and (*d*) matching with costs. The key shows that red indicates the highest transmission and blue the lowest transmission. Horizontal slices through these plots, showing *β* as a function of *h* for particular values of *p*, can be found in the electronic supplementary material, figure S1. The exact forms are: (*a*) 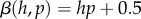, (*b*) 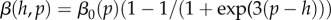 with 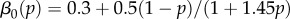, (*c*) 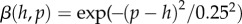, and (*d*) 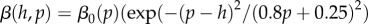 with 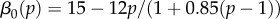. We note that the explicit form of our trade-offs links maximum and minimum trait values through a smooth, polynomial-like curve where the second derivative has constant sign (i.e. no inflections).

It is clearly important to examine the precise nature of the infection function, 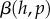, to determine coevolutionary dynamics. Following previous work [5], here we use three key functional forms: ‘universal’ (no specificity), ‘range’ (variation from specialism to generalism) and ‘matching’ (highly specific). These are shown as heat maps in figure 1, where red denotes high transmission rates for combinations of *h* and *p* and blue low transmission. In detail, the universal function is given by2.7
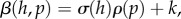
where *k* is a constant giving the minimum value of the infection function. In this case, there is no specificity, as figure 1*a* highlights that parasites retain the same relative ordering of infection rates against any host (see also the electronic supplementary material, figure S1*a*). As such, each host is ‘universally’ more resistant moving from right to left (here 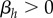, where the subscript denotes differentiation with respect to *h*) and similarly for parasites (*β_p_* > 0).

The range function is given by2.8
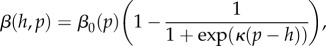
where *κ* is a constant controlling the steepness of the curve. In this case, there is variation in specificity, representing hosts and parasites that range from specialist to generalist. A parasite trade-off, 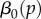, is built into the infection function, so that parasites with a narrow range (low *p*) achieve higher transmission against the least resistant hosts. (The cost of a large range is thus a low transmission rate, and we assume that no further parasite trade-offs to virulence. Including an additional virulence trade-off has no qualitative impact on the results presented here.) The range function, as shown in figure 1*b* (see also the electronic supplementary material, figure S1*b*), therefore includes specificity as for low *h* parasites with low *p* have the highest transmission, but for high *h* parasites with high *p* are the most infectious. Hence, parasites vary in the range of hosts that they can successfully infect, and similarly for host resistance.

For the matching function,2.9
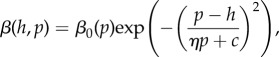
where *η* and *c* are constants controlling the variance of the infection curves. Here, a ‘match’ between host and parasite strains is required for optimal infection, with the transmission rate falling away as they become more distant. This function therefore corresponds to a high degree of specificity between host and parasite. The case where *η* = 0 and 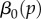 is constant (i.e. there are no costs to the parasite) represents a continuous analogy of matching-allele infection genetics, as shown in figure 1*c* (see also the electronic supplementary material, figure S1*c*; e.g. [5]). When *η* > 0 and we assume costs, the trade-off ensures that parasites with a narrow range achieve higher transmission against their matching hosts relative to parasites with a broader range (again, there is no virulence trade-off in the matching model), as shown in figure 1*d* (see also the electronic supplementary material, figure S1*d*). This is in some sense a hybrid matching-range function, but the maximum transmission of a parasite is not always against the least resistant hosts (cf. figure 1*b*,*d*).

## 3. Results

### (a) Specificity of the infection function

In the electronic supplementary material, §A2, we show that if there are no fitness costs to host resistance or parasite infectivity, then a coevolutionary singular point can never exist for the universal or range functions. As selection now only acts on transmission, the host will always evolve to minimize investment and the parasite to maximize (to bounds of evolution). For the matching function with no costs (i.e. figure 1*c*), there will be a continuum of singular points at *h* *=* *p* none of which are attracting. Under the full assumptions of adaptive dynamics, this will lead to a random walk through trait space. However, if we relax the assumption of mutations occurring rarely, fluctuating selection occurs owing to the ‘trail’ of strains on one side of the current resident. This build-up of strains keeps the host or parasite evolving in the same direction for longer, with reversals in selection owing to one antagonist ‘passing’ the other becoming more rare. We term these ‘stochastic oscillations’, because they are non-deterministic, unstable cycles whose existence depends on the discrete and stochastic assumptions of the simulations. An example of these stochastic oscillations can be found in [5]. For the remainder of this study, we assume that host resistance and parasite infectivity are costly.

We initially consider whether coevolutionary cycles can ever emerge for each infection function. This is particularly important for the universal function because cycles in this model have never been demonstrated (see [4] and [27] for examples of cycles in the range model). To achieve this, initially we simply wish to show that parameters and trade-offs exist that produce a Hopf bifurcation, using a method previously employed to find cycles between parasite virulence and predator population densities [35]. The full analysis is given in the electronic supplementary material, §A2.

**Figure 2.**
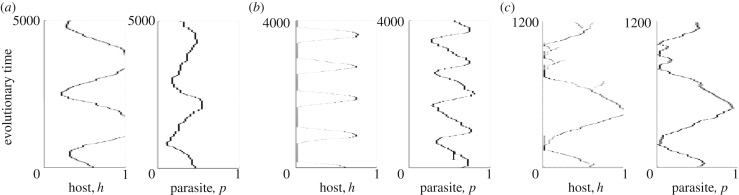
Output from numerical simulations showing the investment in host defence, *h*, and parasite infectivity, *p*, over evolutionary time using the three infection functions from figure 1: (*a*) universal, (*b*) range and (*c*) matching. Simulations were conducted as described in the electronic supplementary material. In (*a*) 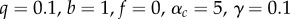, in (*b*) 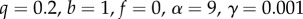 and in (*c*) 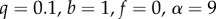 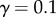. The parasite trade-off in (*a*) is 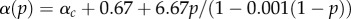 and in (*b*) and (*c*) as given in figure 1. The host trade-offs are (*a*) 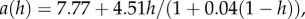 (*b*) 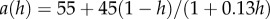 and (*c*) 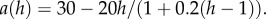 We note that these trade-offs are not subject to the assumptions made when proving the existence of the Hopf bifurcation in the electronic supplementary material (indeed, if we chose trade-offs that satisfied those conditions, we would not see cycles in the simulations).

In the universal model (equation 2.7), cycles will be possible (for some parameters and trade-offs) wherever *k* > 0. However, there is a special case for *k* = 0 (i.e. the minimum value of the infection function is 0), where we show there can never be cycles (see the electronic supplementary material, §A2i). Biologically, this means that cycles in quantitative levels of resistance and infectivity will not occur unless parasites have a non-zero baseline level of transmission, and is owing to the host trait having no impact on parasite selection in this special case (see the electronic supplementary material, §A2i). This explains why in a previous study we found no evidence of coevolutionary cycles with the universal transmission function 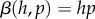 [2]. Figure 2*a* shows numerical simulations of the coevolutionary dynamics for the case where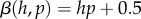 (i.e. *k* > 0) with regular coevolutionary cycles. These cycles lead to regular increases and decreases in quantitative host resistance and parasite infectivity (transmission) and virulence. The cycles arise simply owing to the negative frequency dependence resulting from the epidemiological feedbacks on disease prevalence from the evolution of resistance and infectivity.

We find that a Hopf bifurcation may occur for any form of the range infection function (equation 2.8). The cycles that emerge will be in the respective resistance and infection ranges of hosts and parasites, as demonstrated previously [4,27]. Figure 2*b* shows the output from simulations of the coevolutionary dynamics, once again showing regular cycles.

Assuming costs in the matching model (equation 2.9), we again find that a Hopf bifurcation may always occur. However, in this case, numerical analysis of the system indicated that the Hopf bifurcation is always *subcritical*, meaning that the cycles are unstable (i.e. not attracting) [35,36]. We explored a comprehensive range of parameter sets and trade-offs, but saw no examples of stable coevolutionary cycles in bifurcation diagrams or numerical simulations. Instead, there is generally a bistability such that, under the full assumptions of adaptive dynamics, the system should evolve either to an intermediate singular point or to a minimum. However, as is the case when there were no costs, when the assumptions are relaxed in numerical simulations, we typically see fluctuating selection. An example of these dynamics is shown in figure 2*c* where we see rather irregular oscillations. These are once more non-deterministic, stochastic oscillations. Such stochastic effects are inherent in natural systems; therefore, these oscillations are likely to occur in nature, but we emphasize that these are less regular and predictable than those seen for the universal and range models (cf. figure 2*a*,*b*). Why do such oscillations emerge? In general, the host will always evolve away from the parasite and the parasite will evolve to match the host, leading to a ‘chase’ across phenotypic space (which is again linked to the presence of the ‘trail’ of strains present when mutations are not strictly rare). However, we found that provided the trade-offs are not too strongly decelerating or accelerating, the *h* and *p* nullclines generally remain very close to the main diagonal (*h* *=* *p*), meaning that a small mutation can easily cross the nullclines and reverse the direction of selection, causing the ‘chase’ to go in the other direction (see the electronic supplementary material, figure S6). These repeated crossings of the nullcline by small, finite mutations are what drive the oscillations.

### (b) Host and parasite life-history characteristics

We now explicitly consider the ecological conditions that favour FSD by varying host and parasite life-history traits for each infection function. For the stable cycles, we do this by computing bifurcation diagrams using the numerical continuation software AUTO-07p [37]. For the stochastic oscillations, we examine numerical simulations. In each case, we shall explore the effects of altering (i) resource competition, *q*, and (ii) the virulence, *α*. Plots for the other parameters (*b*, *γ* and *f*) can be found in the electronic supplementary material, figures S2, S3 and S5.

**Figure 3.**
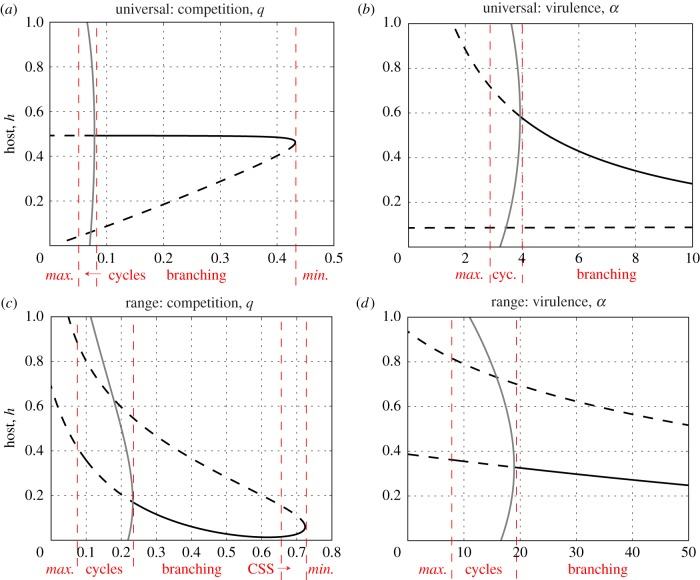
Bifurcation diagrams for (top row) the universal and (bottom row) range models showing the change in behaviour at the singular point as we vary: (*a* and *c*) competition, *q*, and (*b* and *d*) virulence, *α*, in terms of host investment, *h*. Solid black lines denote convergence stable singular points, dashed black lines non-convergence stable singular points (i.e. repellers) and solid grey lines the upper and lower limits of a coevolutionary cycle. The vertical dashed lines separate regions of behaviour as annotated along the bottom of the plots. The *maximize* and *minimize* labels refer to the host's behaviour. In these regions, the parasite either displays the same behaviour or reaches a CSS. Default parameter values are: *q* = 0.1, *b* = 1, *f* = 0 with (*a*) and (*b*) 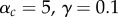, and (*c*) and (*d*) 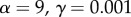 with the trade-offs as given in figures 1 and 2. Again, we note that these trade-offs are not subject to the assumptions made when proving the existence of the Hopf bifurcation. (Online version in colour.)

The behaviour in the universal model as resource competition, *q*, is varied is representative of all of the bifurcation diagrams (figure 3*a*; electronic supplementary material, figure S2). The vertical dashed lines in figure 3 separate the regions of behaviour, as annotated along the bottom. Starting from the right-hand end of figure 3*a*, the trend as *q* is decreased is: no singular point, leading to minimization; the emergence of a pair of singular points through a saddle-node bifurcation (solid line: a branching point; dashed line: a repeller) often leading to branching; a Hopf bifurcation leading to the onset of cycles which increase in size (solid grey line marks that the maximum and minimum values reached on a cycle); the loss of cycles such that both host and parasite maximize (i.e. ARD). We see similar behaviour in figure 3*b* as virulence is varied (although here the saddle-node bifurcation occurs for rates of virulence beyond the domain of this plot). Decreasing values of *q* and *α* lead to increased densities of infectious individuals, and hence, higher encounter rates with susceptible hosts. It is interesting to note that ‘static diversity’ (branching to coexistence) occurs for lower encounter rates than ‘temporal diversity’ (FSD). We conclude that FSD will be promoted in intermediate-large-sized populations (intermediate *q*, low *b* and intermediate *f*), with an intermediate infectious period (intermediate *α*, low *b* and intermediate *γ*). In the electronic supplementary material, §A3 and figure S5, we also show that cycles occur for a range of weakly decelerating trade-offs in both the host and the parasite.

The bifurcation plots for the range model in figure 3*c*,*d* show very similar behaviour to those for the universal model (figure 3*a*,*b*), except that a new behaviour emerges with regions where the singular point is an attracting CSS. The conditions that promote FSD in the range model are qualitatively similar to those in the universal model.

**Figure 4.**
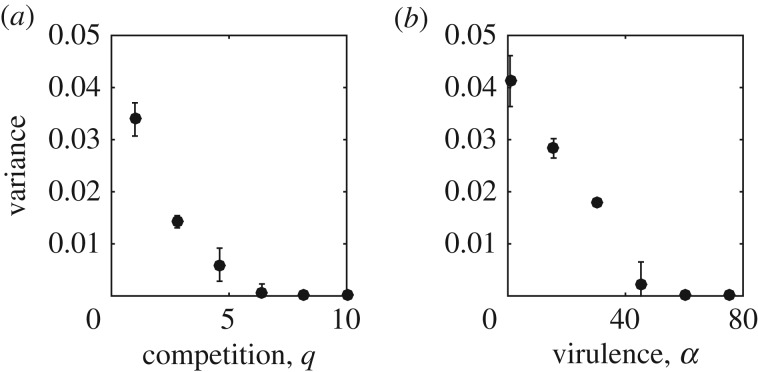
Plots showing the variance in the host trait over the final 20% of numerical simulations, using the matching model for (*a*) competition, *q*, and (*b*) virulence, *α*. A larger variance indicates larger cycles. Zero variance occurs where there is parasite extinction. Parameter values are as in figure 2.

To explore the effects of life-history characteristics on the stochastic oscillations in the matching model, we ran evolutionary simulations and measured the variance in the host trait over the final 20% of each run. A higher variance indicates larger stochastic oscillations (the values where there is zero variance actually relate to parasite extinction). In figure 4 (electronic supplementary material, figure S3), we see a similar pattern to the above results—the variance is greatest in long-lived (low *b*), large populations (low *q*, low *b*) with high infectious periods (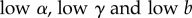).

## 4. Discussion

We have analysed a series of host–parasite coevolutionary models to understand how ecological dynamics, life-history characteristics and the specificity of interactions between hosts and parasites impact FSD. A key finding is that FSD in host resistance and parasite infectivity may occur without the need for any specificity in the interaction between hosts and parasites. When there is specificity, we find that the nature of fluctuating selection is very different in a highly specific matching interaction (akin to matching alleles in that all parasite strains are specialists on respective host strains) compared with when there is variation in the range of specificity (akin to gene-for-gene in that there is variation in specificity such that hosts and parasites vary from specialists to generalists). Therefore, although it is already known that both types of specific infection mechanism can lead to FSD, our models suggest that the nature of the underlying fluctuations is fundamentally different [9]. Finally, we show how both host and parasite characteristics influence the likelihood of fluctuating selection, which allows us to predict the ecological conditions that are most likely to show FSD. This is important because it tells us when fluctuating selection is likely to generate genetic diversity through time [13].

The fact that fluctuating selection can arise without specificity between hosts and parasites is of particular interest because much theoretical and empirical work has focused on identifying the relationship between different types of specificity and FSD rather than considering the potential for FSD in non-specific interactions [7–11,23–25]. We have shown that costs associated with non-specific resistance and infectivity can be sufficient to generate coevolutionary cycles in an eco-evolutionary setting. In principle, these cycles would also be possible in a non-ecological framework where selection is frequency-dependent but not density-dependent, as one could choose fitness functions whereby the selection gradients are never simultaneously zero on a closed trajectory. However, it is realistic to assume that the relative population densities, and thus the prevalence of infection, will vary with changes in host resistance and parasite infectivity. The feedbacks generated by these changes provide a natural route for frequency-dependent selection to operate and generate fluctuations. The drivers of the cycles in both the universal and range models are thus owing to a mixture of frequency dependence (i.e. relative infection rates) and density dependence (i.e. varying population sizes owing to ecological feedbacks). Cycles without specificity have not been described previously, as most studies on FSD have neglected ecological dynamics and feedbacks. Those evolutionary studies that do include ecology have either assumed specificity between host and parasite and not examined universal interactions [16,38–44], or have assumed universal infection but focused on optimal investment or evolutionary branching rather than cycling [1–5,29]. Our work examines models with explicit ecological dynamics and focuses on the potential for FSD both with and without specificity.

Ecology has been shown to drive fluctuating selection in predator–prey systems with specificity [31,45] (although we note that the ‘matching’ function considered in these studies is different from the one used here). However, our work shows that it also occurs in non-specific host–parasite interactions. This result has important relevance to the role host–parasite coevolution may play in shaping host diversity across space and time. When host fitness depends on the frequency of different parasite genotypes, there are predicted to be differences among populations in terms of which host and parasite genotypes are being selected for at a given point in time. Hence, the propensity for fluctuating selection will have impacts on host–parasite local adaptation, as isolated populations are likely to be out of sync with one another [6,19]. There are also implications to the theory surrounding the evolution of sexual reproduction. While evolution of sex studies typically takes a population genetics approach with a few major loci, it has been shown that sex can be beneficial where there are many loci with small additive effects [46]. One common criticism of the Red Queen hypothesis for the maintenance of sex is the lack of highly specific and virulent parasites that are generally assumed to be necessary for FSD [47]. Our work suggests that these restrictive assumptions could be relaxed; future theory must test whether selection for sex can be generated in the absence of specificity and for parasites with only intermediate levels of virulence.

While we found that FSD could occur across all of the interactions we considered, we found that the nature of the cycles is fundamentally different. We have shown that both the universal and range infection functions can lead to regular, deterministic cycles when there are costs. For the universal function, this leads to fluctuations in the transmission rate, while for the range function the fluctuations are between pure generalists and pure specialists. However, when there is a matching function, we found that stable deterministic cycles do not exist. Instead, we have shown that oscillations occur driven by the inherent incompatibility of the optimal host and parasite strategies. This result is in accordance with models of matching alleles in continuous time, which have shown only damped cycles rather than deterministic stable limit cycles [43,48]. This result also relates to the idea of ‘stochastic persistence’ [49], because regular input of mutations (i.e. faster than a full separation of ecological and evolutionary timescales) is essential for the cycles to be sustained. There are a number of implications to these different types of cycles. The deterministic cycles generated by the universal and range models are more regular and consistent, making their behaviour more predictable. By contrast, the stochastic oscillations of the matching interaction tend to be irregular and vary in period and amplitude, making their behaviour unpredictable. Stochastic fluctuations may also be less robust to changes in assumptions about mutation and standing variation. Distinguishing between these two forms of cycles empirically would be challenging owing to environmental variation, but if FSD can be observed over multiple cycles, evidence of regularity could be looked for. An exciting question that thus emerges is whether the inherent differences among the fluctuating dynamics observed across infection interactions might support different levels of genetic diversity within and among populations. It is yet unclear whether cycles generated under a specialist–generalist continuum (i.e. range or gene-for-gene) framework can be considered equivalent to those generated under a purely specialist (i.e. matching) framework.

By including explicit ecological dynamics in our models, we have been able to assess how host and parasite life-history characteristics impact the potential for FSD. We have found that, no matter the infection function, cycles are most likely when hosts are long-lived and exist at high, but not the highest, densities. These results suggest that cycles are promoted when encounter rates are reasonably high. When encounter rates are low, so too is the potential for infection; therefore, selection for costly host resistance is likely to be limited. At the other extreme, if encounter rates are very high, then there will be considerable selection for resistance, leading to an ARD. It is in between these two extremes when cycles are most likely to occur. These results emphasize the role ecology plays in driving FSD in our models, because cycles only arise for certain regions of parameter space. Empirical studies in bacteria–phage systems agree with the predictions from our models, with environmental conditions that increase host–parasite encounter rates, causing a shift from FSD to ARD [26–28]. This pattern is consistently seen in the stochastic oscillations from the matching model as well as the stable cycles of the universal and range models, suggesting that this parameter dependency is robust.

Our models have demonstrated that there are a wide range of interactions between hosts and parasites that can lead to fluctuating selection. We require that there are costs to resistance and infectivity to produce deterministic cycles in range or universal models, consistent with previous theory showing that costs are necessary but not sufficient for FSD to occur in gene-for-gene systems [3]. However, highly specific matching interactions produce stochastic oscillations. Our models are novel in that they demonstrate that specificity is not required for fluctuating selection to occur. Both the host life-history and the disease characteristics that promote FSD are consistent across all the different infection interactions. We can therefore make robust predictions for the types of host–parasite interactions that are most likely to lead to coevolutionary cycles. We note that the timescale of the cycles seen in our models is somewhat slower than those seen in classic gene-for-gene or matching-allele models. This is because we assume a separation of ecological and evolutionary timescales, whereas the genetic models are essentially at an ecological timescale with multiple competing strains. The cycles considered here are purely at the evolutionary timescale, with the population dynamics always being at, or close to (in simulations), an equilibrium. We also note that our methods assume a large number of loci with small additive effects, as opposed to classic population genetics models, which generally assume a small number of loci and epistasis between them. Future work will address when the discreteness that arises from a smaller number of loci has a significant effect on the results, but without a detailed understanding of the genetic basis of a particular interaction the quantitative assumption gives general insights.

Empirical evidence from a number of host–parasite systems indicates that fluctuating selection is a common form of coevolutionary dynamics. Several studies have reported indirect evidence of FSD (or host–parasite relationships capable of FSD) based on phylogenetic data (e.g. *Arabidopsis* plants and *Pseudomonas* bacteria [50]), highly specific genetic interactions (e.g. sticklebacks and trematodes, [51]), or high levels of polymorphism in immune genes (e.g. in the vertebrate major histocompatibility complex, [52]). Direct evidence of FSD primarily comes from time-shift experiments [53] between crustaceans and bacteria [54], water snails and trematodes [55], and bacteria and phages [26–28,56]. The predictions from our models therefore have wide relevance within coevolutionary host–parasite systems. Given the ubiquity of ecological feedbacks and the diversity of different infection interactions, our work emphasizes the considerable potential for host–parasite coevolution to generate fluctuating selection.

## Supplementary Material

Mathematical details and supplementary figures

## Supplementary Material

Simulation code

## Data accessibility

C++ code used for the simulations can be found in the electronic supplementary material.
