# Supplementary material for: Host–parasite fluctuating selection in the absence of specificity
Source: Proc Biol Sci. 2017 Nov 1;284(1866):20171615. doi: 10.1098/rspb.2017.1615 (PMC5698645; doi:10.1098/rspb.2017.1615)
Supplement: Mathematical details and supplementary figures [file rspb20171615supp1.pdf]

Supplementary Information

A. Detailed description of analytic methods and results

A1. Model and methods

As stated in the main text, we base the host-parasite population dynamics on a classic SIS model as follows:

$$(S1)\ \frac{dS}{dt} = (a - q(S + I))(S + fI) - bS - \beta SI + \gamma I$$

$$(S2)\ \frac{dI}{dt} = \beta SI - (b + \alpha + \gamma)I.$$

with the parameters as described in the main text. *For consistency, in this SI we refer to the equation numbers from within this document, and not to those in the main text.* The methods used below not only build on classic adaptive dynamics theory (Metz et al., 1996; Marrow et al., 1996; Dieckmann & Law, 1996; Geritz et al., 1998) but also the related tools of critical function analysis (de Mazancourt & Dieckmann, 2004; Bowers et al., 2005; Kisdi, 2006) and, in particular, a recent study focussed on finding cycles between parasite virulence and predator densities (Kisdi et al., 2013).

Using the adaptive dynamics framework to consider evolution, the success or otherwise of an invading mutant depends on its invasion fitness, given by its exponential growth rate when rare (Metz et al., 1996; Geritz et al., 1998). For a mutant host we can calculate this from the dominant eigenvalue of the mutant equations' Jacobian as,

(S3) $s(\bar{h}; h, p) = \lambda_+ = \frac{T}{2} + \sqrt{T^2 - 4D}/2$

where,

(S3) $T = a(\bar{h}) - q(\hat{S} + \hat{I}) - 2b - \beta(\bar{h}, p)\hat{I} - \alpha(p) - \gamma$

(S4) $D = [a(\bar{h}) - q(\hat{S} + \hat{I}) - b - \beta(\bar{h}, p)\hat{I}][-b - \alpha - \gamma]$

$-[\gamma + f(a(\bar{h}) - q(\hat{S} + \hat{I}))]\beta(\bar{h}, p)\hat{I}$

and here (and below), $\hat{I}$ and $\hat{S}$ represent the steady state values at the resident equilibrium. We note, as stated in the main text, that in the case *f*=0, $\gamma = 0$ this simplifies to,

(S6) $s(\bar{h}; h, p) = a(\bar{h}) - q(\hat{S} + \hat{I}) - b - \beta(\bar{h}, p)\hat{I}$

and we will use this expression from here on in our algebraic analysis. For the parasite we can derive its fitness from the growth rate of a mutant in the infected class as,

(S7) $r(\bar{p}; h, p) = \beta(h, \bar{p})\hat{S} - b - \alpha(\bar{p}) - \gamma.$

Assuming *f*=0, $\gamma = 0$, the coevolutionary dynamics are described by,

(S8) $\frac{dh}{dt} = \varphi^h S \left.\frac{\partial s}{\partial \bar{h}}\right|_{\bar{h}=h} = \varphi^h \hat{S}[a_{\bar{h}} - \beta_{\bar{h}}\hat{I}]$

$$\text{(S9)}\ \frac{dp}{dt} = \varphi^p \frac{\partial r}{\partial \bar{p}}\bigg|_{\bar{p}=p} = \varphi^p \hat{I}[\beta_{\bar{p}}\hat{S} - \alpha_{\bar{p}}]$$

where subscripts denote derivatives and φ^h and φ^p controls the respective speed of evolution between host and parasite (which is the product of the mutation rate and variance and a factor of 1/2). We will assume throughout that $\varphi^h = \varphi^p = 1$ to simplify parts of the analysis.

Coevolution will continue until either the two selection gradients are simultaneously zero (i.e. there is no longer directional selection on either species) and a coevolutionary 'singular point' has been reached (h^*,p^*), or until a maximum or minimum trait has been reached (due to physiological constraints in the host or parasite – note that at such point the fitness gradients are not necessarily zero but further evolution in one direction is prevented), or it may continually cycle. At a singular point, the coevolutionary behavior depends on a number of second-order properties (see Geritz et al., 1998). The point is *evolutionarily stable* (ES) if nearby mutants cannot invade the resident strategy; it is *convergence stable* (CS) if the point is locally attracting; and it is *mutually invadable* (MI) if both mutant and resident strains have positive invasion fitness. ES is given by the second-order term, for example in the host,

$$\text{(S10)}\ \frac{\partial^2 s}{\partial \bar{h}^2}\bigg|_{\bar{h}=h=h^*} < 0$$

while convergence stability for a coevolutionary model is determined by the 2x2 Jacobian (Marrow et al., 1996),

$$(S11)\ J = \begin{pmatrix} \varphi^h \hat{S}\left[\frac{\partial^2 s}{\partial \bar{h}^2} + \frac{\partial^2 s}{\partial \bar{h}\partial h}\right] & \varphi^h \hat{S}\frac{\partial^2 s}{\partial \bar{h}\partial p} \\ \varphi^p \hat{I}\frac{\partial^2 r}{\partial h \partial \bar{p}} & \varphi^p \hat{I}\left[\frac{\partial^2 r}{\partial \bar{p}^2} + \frac{\partial^2 r}{\partial \bar{p}\partial p}\right] \end{pmatrix}$$

evaluated at the singularity, and mutual invadibility by the mixed derivative, for example in the host,

$$(S12)\ \frac{\partial^2 s}{\partial \bar{h}\partial h}\bigg|_{\bar{h}=h=h^*} < 0$$

The four cases of particular interest, as described in the main text are: a *Continuously Stable Strategy* (ES and CS), an *evolutionary branching point* (CS and MI but not ES), *coevolutionary cycles* (loss of CS through a supercritical Hopf bifurcation), and *maximisation/minimisation* of one or both species to bounds of evolution (non-CS but not cycling).

The key infection functions we use are shown in figure 1 in the main text. In figure S1 we also show these functions as 'snapshots' of infection for specific parasite types against all hosts. To recap, the general forms are:

i) Universal:

$$(S13)\ \beta(h,p) = \sigma(h)\rho(p) + k$$

ii) Range:

$$(S14)\ \beta(h,p) = \beta_0(p)\left(1 - \frac{1}{1+\exp\big(\kappa(p-h)\big)}\right)$$

iii) Matching:

(S15) $\beta(h,p) = \beta_0(p)exp\left(-\left(\frac{p-h}{\eta p+c}\right)^2\right)$

A2. Results

No costs

We first consider the case where both the host and parasite can evolve to change the transmission term without incurring costs. Without costs to either the host or parasite (including both forms of parasite trade-off considered here) the respective fitness gradients found using equations (S8) and (S9) are given by

(S16) $\left.\frac{\partial s}{\partial \bar{h}}\right|_{\bar{h}=h} = -\beta_{\bar{h}}\hat{I}$

(S17) $\left.\frac{\partial r}{\partial \bar{p}}\right|_{\bar{p}=p} = \beta_{\bar{p}}\hat{S}$

Therefore a coevolutionary singular point can only exist if $\beta_{\bar{h}} = \beta_{\bar{p}} = 0$. By definition this can never be true for a Universal or Range function (since $\beta_h \neq 0$ in both cases) and would only be true for the specific Matching function when h=p and $\beta_0(p) = \beta_0$ is a constant (e.g. figures 1c, S1c). In fact, in this case there will be a continuum of such singular points, none of which is convergence stable. Under the full assumptions of adaptive dynamics, this will lead to a random walk through trait space. However, if we assume that there is not a complete separation of timescales such that new mutations can appear before the system has reached its dynamic attractor, cyclic behavior will be seen due to the 'trail' of strains on one side of the current resident. An example of these '*stochastic cycles*' from simulations of the coevolutionary process can be found in Boots et al., 2014.

With costs

Here we initially show that any generic infection function, including all three of our example cases, may yield a Hopf bifurcation (a critical point where a system's stability switches from an equilibrium to a limit cycle) when there are costs to resistance and/or infectivity. We consider the convergence stability of the singular point where the two fitness gradients (S8) and (S9) are simultaneously zero. This is governed by the eigenvalues of the Jacobian, J, of the system (Marrow et al., 1996 and see above),

$$\text{(S18)}\ J = \begin{pmatrix} \hat{S}[a_{\bar{h}\bar{h}} - \beta_{\bar{h}\bar{h}}\hat{I} - \beta_{\bar{h}}\hat{I}_h] & \hat{S}[-\beta_{\bar{h}p}\hat{I} - \beta_{\bar{h}}\hat{I}_p] \\ \hat{I}[\beta_{h\bar{p}}\hat{S} + \beta_{\bar{p}}\hat{S}_h] & \hat{I}[\beta_{\bar{p}\bar{p}}\hat{S} - \alpha_{\bar{p}\bar{p}} + \beta_{\bar{p}}\hat{S}_p] \end{pmatrix}$$

where subscripts denote derivatives, and hereafter evaluation at the singular point is intended but not made explicit. Let us for now assume that there is no simple decomposition of $\beta(h,p)$. The characteristic equation of this Jacobian reveals that the eigenvalues of the singular point are,

$$\text{(S19)}\ \lambda = \frac{B}{2} \pm \frac{\sqrt{B^2 - 4C}}{2}$$

where

$$\text{(S20)}\ B = tr(J) = \hat{S}\left[a_{\bar{h}\bar{h}} - \beta_{\bar{h}\bar{h}}\hat{I} - \beta_{\bar{h}}\hat{I}_h\right] + \hat{I}[\beta_{\bar{p}\bar{p}}\hat{S} - \alpha_{\bar{p}\bar{p}} + \beta_{\bar{p}}\hat{S}_p]$$

$$\text{(S21)}\ C = \det(J) = \hat{S}\hat{I}\big(\left[a_{\bar{h}\bar{h}} - \beta_{\bar{h}\bar{h}}\hat{I} - \beta_{\bar{h}}\hat{I}_h\right]\left[\beta_{\bar{p}\bar{p}}\hat{S} - \alpha_{\bar{p}\bar{p}} + \beta_{\bar{p}}\hat{S}_p\right] + [\beta_{\bar{h}p}\hat{I} +$$

$\beta_{\bar{h}}\hat{I}_p][\beta_{h\bar{p}}\hat{S} + \beta_{\bar{p}}\hat{S}_h])$

For a Hopf bifurcation to occur, the two eigenvalues must have Im(λ) non-zero with Re(λ) simultaneously equal to zero; This occurs when B=0 and C>0. At this point we simply wish to show that parameters and trade-offs exist that produce a Hopf bifurcation. We can therefore fix our trade-offs to take certain forms. We shall explore how the potential for cycles depends on the trade-off shape later in the main text. In particular, no matter the sign of each of the derivatives in B, one or more of the trade-off curvatures can always be chosen such that B=0; for example $a_{\bar{h}\bar{h}} = \beta_{\bar{h}\bar{h}}\hat{I} + \beta_{\bar{h}}\hat{I}_h$; $\alpha_{\bar{p}\bar{p}} = \beta_{\bar{p}\bar{p}}\hat{S} + \beta_{\bar{p}}\hat{S}_p$ is a particularly useful choice, though clearly many other combinations exist that satisfy $B=0$. It therefore remains to show that $C > 0$ is possible in the neighbourhood of $B = 0$. Since from (S20) at $B = 0$ we have $\hat{I}[\beta_{\bar{p}\bar{p}}\hat{S} - \alpha_{\bar{p}\bar{p}} + \beta_{\bar{p}}S_p] = -\hat{S}[a_{\bar{h}\bar{h}} - \beta_{\bar{h}\bar{h}}\hat{I} - \beta_{\bar{h}}\hat{I}_h]$, we can now write,

(S22) $C = \hat{S}\hat{I}\left(-\frac{I}{S}[\beta_{\bar{p}\bar{p}}\hat{S} - \alpha_{\bar{p}\bar{p}} + \beta_{\bar{p}}\hat{S}_p]^2 + [\beta_{\bar{h}p}\hat{I} + \beta_{\bar{h}}\hat{I}_p][\beta_{h\bar{p}}\hat{S} + \beta_{\bar{p}}\hat{S}_h]\right)$

Again, though, we can always choose curvatures that make the first term zero without jeopardising the value of $B = 0$; in particular our choice above of $a_{\bar{h}\bar{h}} = \beta_{\bar{h}\bar{h}}\hat{I} + \beta_{\bar{h}}I_h$; $\alpha_{\bar{p}\bar{p}} = \beta_{\bar{p}\bar{p}}\hat{S} + \beta_{\bar{p}}\hat{S}_p$ would satisfy both conditions. It therefore remains for us to show that

(S23) $C = \hat{S}\hat{I}[\beta_{\bar{h}p}\hat{I} + \beta_{\bar{h}}\hat{I}_p][\beta_{h\bar{p}}\hat{S} + \beta_{\bar{p}}\hat{S}_h]$>0

is possible. By considering the derivatives of the *S* and *I* equilibrium densities (i.e. the solutions to (S1)-(S2)), and now explicitly assuming evaluation at the singular point, we find that (S23) becomes

$$\text{(S24)}\quad C = \hat{S}^2\hat{I}^2\left[\beta_{hp} - \frac{\beta_h\beta_p}{q+\beta}\right]\left[\beta_{hp} - \frac{\beta_h\beta_p}{\beta}\right] > 0$$

Clearly for small q (low competition, meaning large population sizes) this condition will be satisfied. Thus we have established that, for suitable cost structures, there may be eigenvalues with zero real part and non-zero imaginary parts, indicating the existence of a Hopf bifurcation and the potential for cycles. It is important to note that this is the point at which cycles emerge. Once this point is passed through, further changes to the trade-offs or parameters will lead to the cycles growing in size (with the eigenvalues at the singular point being complex with positive real part). Thus, while the trade-off curvatures are required to obey certain conditions at the Hopf bifurcation, coevolutionary cycles will occur for a wider range of conditions (see §A3 and figure S5 of this SI). Moreover, maintaining the choice of curvature described above, if we choose the gradients and curvatures such that C=0, then we have zero trace and zero determinant of our Jacobian which corresponds to a Bogdanov-Takens point, a co-dimension two bifurcation which is the meeting of saddle-node and Hopf bifurcation curves (Kuznetsov, 1995, Kisdi et al., 2013). At such a point there necessarily emerges a homoclinic orbit, bounding the region of the cycles. In particular, once the cycles grow to such a size that they pass the homoclinic orbit, there will be no convergence stable singular points or stable coevolutionary

cycles remaining in the system. The system would therefore evolve to maximum or minimum investment by the host or parasite depending on the positioning of the nullclines. Also, the existence of the saddle-node bifurcation suggests parameter values exist where there are no singular points present.

We now apply the general condition we have calculated to each of the example infection functions we introduced in figure 1.

i) Universal

In the case of universal transmission with $\beta(h,p) = \sigma(h)\rho(p) + k$, we are able to further simplify the expression in equation (S24) since $\beta_{hp} = \sigma_h\rho_p$; $\beta_h = \sigma_h\rho$; $\beta_p = \sigma\rho_p$ (recall subscripts denote derivatives), meaning this becomes

(S25) $$C = \hat{S}^2\hat{I}^2\left[\sigma_h(h)\rho_p(p)\right]^2\left[\frac{k}{\sigma\rho+k}\right]\left[\frac{k+q}{\sigma\rho+k+q}\right] > 0$$

(assuming that our trade-off curvatures are such that B (S20) and the first term of C (S21) are both zero as described above). Wherever $k > 0$ (S25) is necessarily positive so cycles will always be possible. We see that in the special case that k=0 we have C=0 and there can be no cycles in the system. In fact, it can be shown that when k=0 the bottom-left entry in the Jacobian (S18) is necessarily zero, ensuring that there are no cycles even without making assumptions on the trade-offs as above. To show this we note that by differentiating at the population dynamics equilibrium we have $S_h = -\beta_h S/\beta$, meaning the bottom-left entry in (S18) becomes,

(S26) $\hat{I}[\beta_{hp}S - \beta_h\beta_p S/\beta]$.

Now substituting the general universal infection function $\beta = \sigma(h)\rho(p) + k$ and its derivatives in to (S26) we have,

(S27) $\hat{I}\left[\sigma_h\rho_p S - \frac{\sigma_h\rho_p\sigma(h)\rho(p)S}{\sigma(h)\rho(p)+k}\right]$,

which vanishes when k=0. Because of this we know that the eigenvalues must be real, hence there can be no cycling, irrespective of any assumptions on the trade-offs. We note that the interpretation of this entry being zero is that the host trait is having no impact on the parasite selection gradient.

(ii) Range

The condition for cycling in the range model reduces considerably compared to (S24) since $\alpha(p) = \alpha$ is a constant. This means not only do we know that $\alpha_{pp} = 0$, but also that at the singular point we must have $\beta_p = 0$ from (S9). As such, a choice of $\beta_{pp} = 0$ means that the bottom-right entry in the Jacobian (S18) is necessarily zero, and only a condition remains to be taken on the host's trade-off is required to let B=0. Additionally, substituting these zero values in to equation (S24), in particular noting again that $\beta_p = 0$, means that the condition for cycling reduces to,

(S28) $C = \hat{S}^2\hat{I}^2\beta_{hp}^2 > 0$

which is always true and so we confirm that a Hopf bifurcation may occur for any form of this infection function.

(iii) *Matching*

Here we again assume that virulence is not involved in the parasite trade-off. This means that the condition for cycling is identical to equation (S26) for range, which is always true so a Hopf bifurcation can occur for any form of this infection function. However, when analysing this model we found that the Hopf bifurcation is always *subcritical* meaning that the cycles that arise are unstable. The complexity of the model prevented us from proving this analytically, but we plotted bifurcation diagrams and conducted numerical simulations for a comprehensive range of parameters and found that this was always the case.

A3. Host and Parasite Trade-offs

When proving the existence of a Hopf bifurcation in the earlier section, we fixed our trade-offs such that the curvatures at the co-singular point took particular values. Here we explore how the potential for cycles depends on the trade-off shapes, and in particular show that cycles are not limited to a restrictive and/or unrealistic set of trade-offs. In figure S5 we show bifurcation diagrams as the parameter that controls the curvature in the respective trade-off functions (see figure legend) is varied for the universal model. We note that a value of zero gives a linear trade-off. It is clear from these diagrams that cycles occur for a reasonable range of near-linear trade-off shapes. In particular cycles occur for weakly decelerating trade-offs for both host and parasite. Similar results occur for the range model.

References found only in the SI

De Mazancourt, C. & Dieckmann, U. (2004). Trade-off geometries and frequency-dependent selection. *Am. Nat.*, 164:765-778.

Bowers, R., Hoyle, A., White, A. and Boots, M. (2005). The geometric theory of adaptive evolution: trade-off and invasion plots. *J. Theor. Biol*, 233:363-377.

Kisdi (2006). Trade-off geometries and the adaptive dynamics of two coevolving species. *Evol. Ecol. Res.*, 8:959-973.

B. Numerical simulation methods

The simulation code used is available as a supplementary file. The simulations were conducted in the C++ programming language. Arrays of N=50 host ($h=0:1$) and parasite ($p=0:1$) types were initialised, and the densities of all but one host and parasite type set to 0. The following algorithm then applied in each evolutionary time-step:

- The population dynamics of all types were numerically solved using a 4th order Runge-Kutta solver for a large enough number of steps (8000) that the system is nearby its dynamic attractor.
- Any types whose density was below a low threshold of 0.005 were assumed extinct and their densities set to 0.
- A new (mutant) host or parasite type was generated one strain 'up' or 'down' from a resident type. Often multiple strains would still be present as the population dynamics had not yet reached the dynamic attractor. In this case, the 'resident' (i.e. the strain producing the mutant) was chosen probabilistically based on the relative densities

(i.e. if two strains were present at densities of 3 and 2 respectively, there would be a 60% chance of the first strain generating the mutant and a 40% chance of the second strain). The density of the mutant strain was set at 10% of the resident (if the strain already had a positive density it was increased by this amount).

- The initial invasion success of the mutant was checked against a demographic stochasticity function (see Dieckmann & Law, 1996). This function causes extinction of mutant strains with negative or positive but small initial growth rates. With a probability inversely proportional to fitness (with negative fitness meaning 0 probability), that mutation event does not occur. This ensures that the relative speeds of evolution of the two coevolving species match with the analytical approach.

This evolutionary time-step algorithm was then repeated. The densities were recorded at the end of each run of the population dynamics.

SI Figures

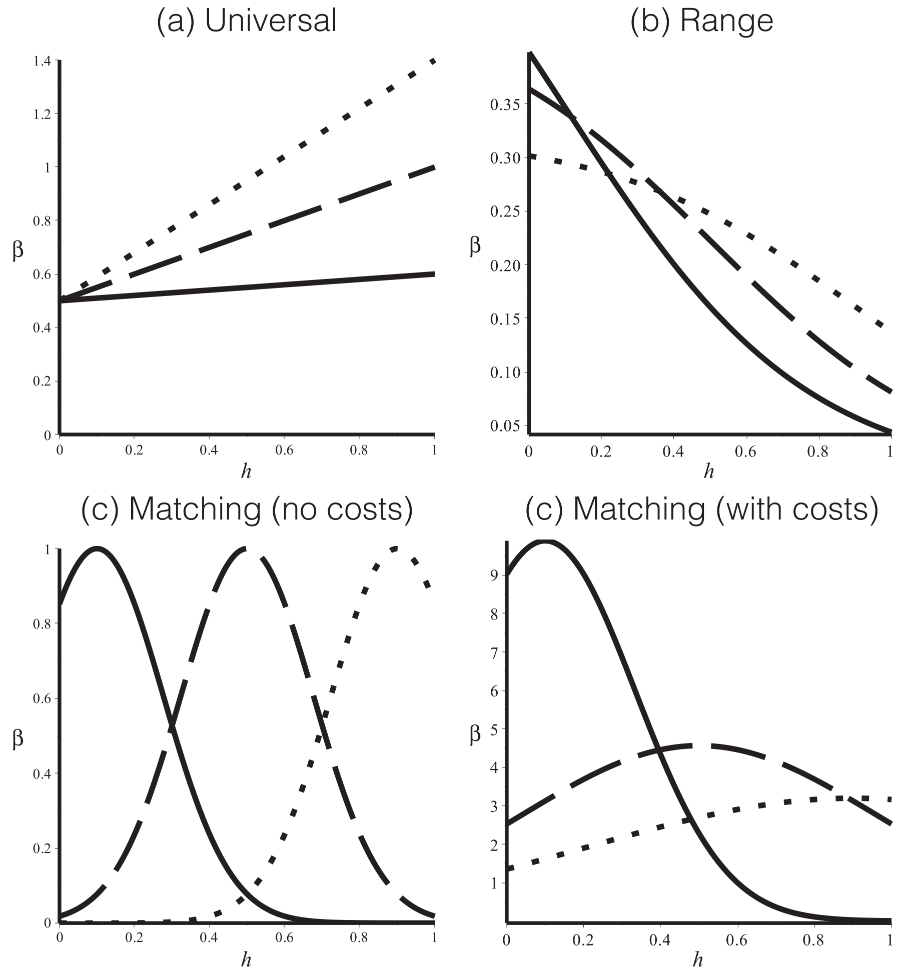

Figure S1

Plots showing snapshots of transmission, β, for specific parasite strains, p, (solid line is for $p=0.1$, dashed line $p=0.5$ and dotted line $p=0.9$) against all host strains, h, for (a) Universal, (b) Range, (c) Matching without costs and (d) Matching with costs. The exact forms are: (a) $\beta(h,p) = hp + 0.5$, (b) $\beta(h,p) = \beta_0(p)(1 - 1/(1 + \exp(3(p-h))))$ with $\beta_0(p) = 0.3 + 0.5(1-p)/(1 + 1.45p)$, (c) $\beta(h,p) = \exp(-(p-h)^2/0.25^2)$, (d) $\beta(h,p) = \beta_0(p)(\exp(-(p-h)^2/(0.8p + 0.25)^2)$ with $\beta_0(p) = 15 - 12p/(1 + 0.85(p-1))$.

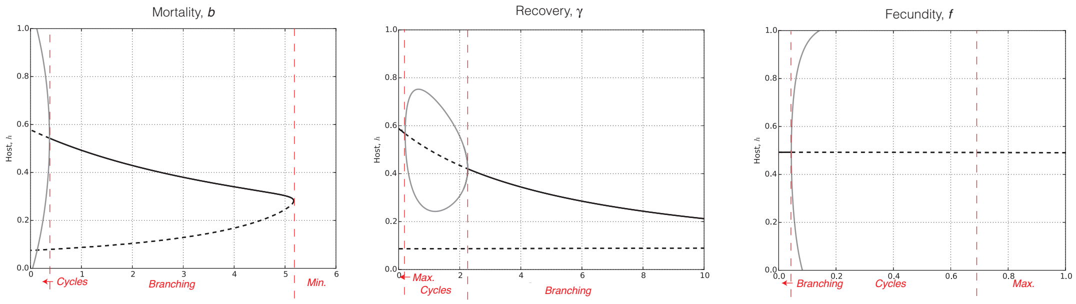

Figure S2

Bifurcation diagrams for the universal model showing the change in behavior at the singular point as we vary: (a), mortality, b, (b) recovery, γ, and (c) infected fecundity, f, in terms of host investment, h. Solid black lines denote convergence stable singular points, dashed black lines non-convergence stable singular points (i.e. repellers) and solid gray lines the upper and lower limits of a coevolutionary cycle. The red vertical dashed lines separate regions of behavior as annotated along the bottom of the plots. Default parameter values are as of figure 3 in the main text. (We note here that the general results are not restricted to assuming high levels of sterilization. Cycles in the universal model with our default parameters occur for intermediate levels of sterility (see panel (c)), but we found that they could still occur up to f=1 for other parameter sets (results not shown).)

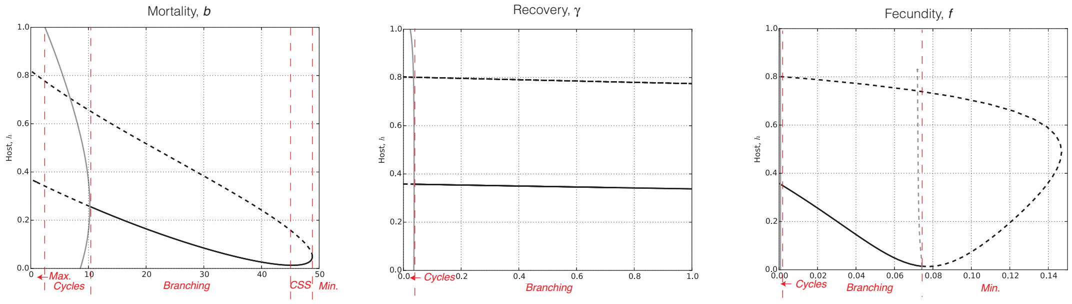

Figure S3

Bifurcation diagrams for the range model showing the change in behavior at the singular point as we vary: (a), mortality, b, (b) recovery, γ, and (c) infected fecundity, f, in terms of host investment, h. Solid black lines denote convergence stable singular points, dashed black lines non-convergence stable singular points (i.e. repellers) and solid gray lines the upper and lower limits of a coevolutionary cycle. The red vertical dashed lines separate regions of behavior as annotated along the bottom of the plots. Default parameter values are as of figure 2 in the main text.

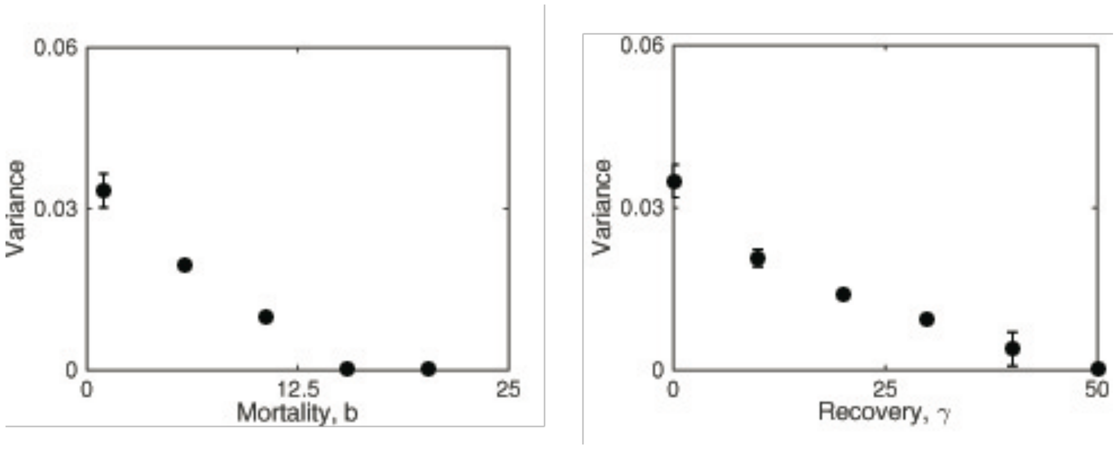

Figure S4

Plots showing the variance in the host trait over the final 20% of numerical simulations, using the matching model for (a) mortality, *b*, and (b) recovery, γ. A larger variance indicates larger cycles. Zero variance occurs where there is parasite extinction. Parameter values are as of figure 4 in the main text.

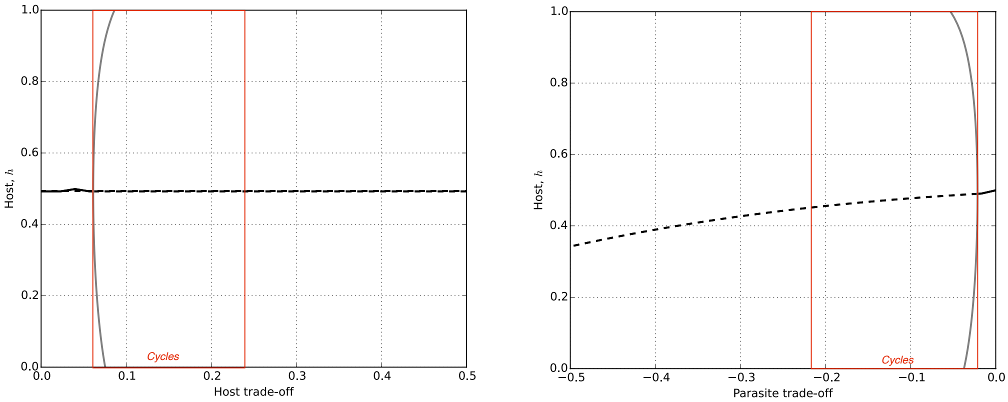

Figure S5

Bifurcation diagrams as (left) the host trade-off shape and (right) parasite trade-off shape are varied. The trade-offs used are $a(h) = 7.77 + 4.51h/(1 + \vartheta_h(1-h))$ and $\alpha(p) = \alpha_c + 0.67 + 6.67p/(1 - \vartheta_p(1-p))$, and it is the ϑ parameters which are varied. Solid black lines denote convergence stable singular points, dashed black lines non-convergence stable singular points (i.e. repellers) and solid gray lines the upper and lower limits of a coevolutionary

cycle. The red vertical dashed lines highlight the regions that generate cycles. Default parameter values are as of figure 3 in the main text.

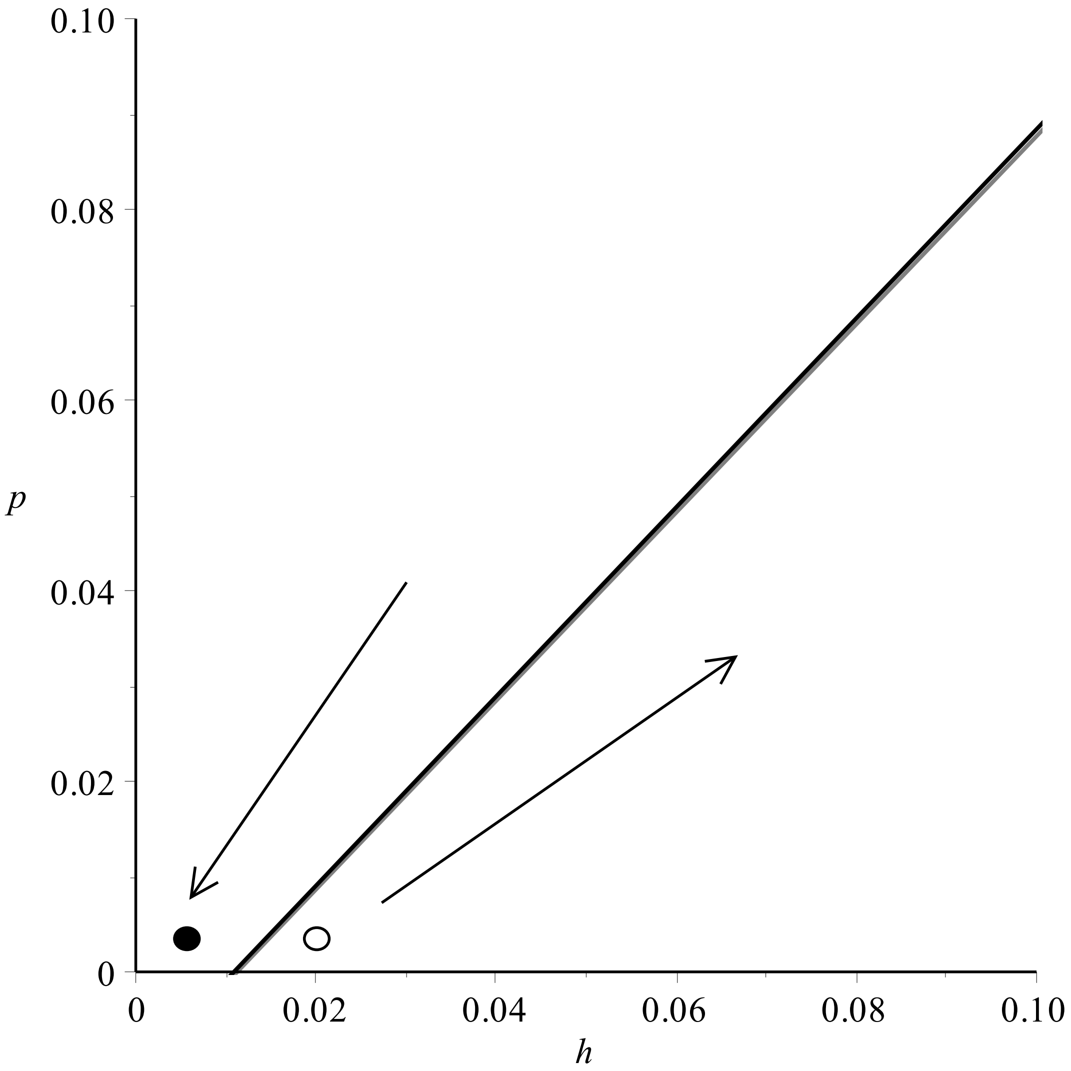

Figure S6

The deterministic adaptive dynamics for the matching model in the phase space of *h* and *p*. The host nullcline is shown in black and the parasite nullcline in gray. Above both nullclines selection is to reduce investment in both the host and parasite, which would lead to minimal investment. However, the nullclines are suitably close to the main diagonal that small mutations allow strains to appear below the nullclines, where selection is to increase investment.
